# pyMeSHSim: an integrative python package for biomedical named entity recognition, normalization, and comparison of MeSH terms

**DOI:** 10.1186/s12859-020-03583-6

**Published:** 2020-06-18

**Authors:** Zhi-Hui Luo, Meng-Wei Shi, Zhuang Yang, Hong-Yu Zhang, Zhen-Xia Chen

**Affiliations:** 1 Hubei Key Laboratory of Agricultural Bioinformatics, College of Life Science and Technology, Huazhong Agricultural University, Wuhan, Hubei 430070 PR China; 2 College of Biomedicine and Health, Huazhong Agricultural University, Wuhan, Hubei 430070 PR China; 3 Hubei Key Laboratory of Agricultural Bioinformatics, College of Informatics, Huazhong Agricultural University, Wuhan, Hubei 430070 PR China

**Keywords:** MeSH, UMLS, Named entity recognition, Semantic similarity, Supplementary concept records, Disease

## Abstract

**Background:**

Many disease causing genes have been identified through different methods, but there have been no uniform annotations of biomedical named entity (bio-NE) of the disease phenotypes of these genes yet. Furthermore, semantic similarity comparison between two bio-NE annotations has become important for data integration or system genetics analysis.

**Results:**

The package pyMeSHSim recognizes bio-NEs by using MetaMap which produces Unified Medical Language System (UMLS) concepts in natural language process. To map the UMLS concepts to Medical Subject Headings (MeSH), pyMeSHSim is embedded with a house-made dataset containing the main headings (MHs), supplementary concept records (SCRs), and their relations in MeSH. Based on the dataset, pyMeSHSim implemented four information content (IC)-based algorithms and one graph-based algorithm to measure the semantic similarity between two MeSH terms. To evaluate its performance, we used pyMeSHSim to parse OMIM and GWAS phenotypes. The pyMeSHSim introduced SCRs and the curation strategy of non-MeSH-synonymous UMLS concepts, which improved the performance of pyMeSHSim in the recognition of OMIM phenotypes. In the curation of 461 GWAS phenotypes, pyMeSHSim showed recall > 0.94, precision > 0.56, and F1 > 0.70, demonstrating better performance than the state-of-the-art tools DNorm and TaggerOne in recognizing MeSH terms from short biomedical phrases. The semantic similarity in MeSH terms recognized by pyMeSHSim and the previous manual work was calculated by pyMeSHSim and another semantic analysis tool *meshes*, respectively. The result indicated that the correlation of semantic similarity analysed by two tools reached as high as 0.89–0.99.

**Conclusions:**

The integrative MeSH tool pyMeSHSim embedded with the MeSH MHs and SCRs realized the bio-NE recognition, normalization, and comparison in biomedical text-mining.

## Background

Biomedical named entity (bio-NE) recognition, normalization, and comparison are fundamental tasks for extracting and utilizing valuable biomedical information from textual data. They are important to disease diagnosis [1], drug repositioning [2], over-representation analysis [3], and genetic analysis [4]. These functions are realized by identifying key entities in unstructured texts, mapping identified entities to a controlled vocabulary, and measuring the semantic similarity between the vocabulary terms [5].

Medical Subject Heading (MeSH) is a controlled vocabulary that can be used in bio-NE recognition, normalization and comparison [6]. It consists of three main record types including descriptor records, qualifier records, and supplementary concept records (SCRs). MeSH is curated by the National Library of Medicine (NLM) and serves as the index system in PubMed/MEDLINE and other NLM databases. Since 2002, NLM has used Medical Text Indexer (MTI) to provide indexing recommendations based on MeSH in the bio-NE recognition for literatures [7]. Due to its precise literature annotations, MeSH has become more and more popular for normalizing bio-NEs such as disease names, in medical and genetic public databases [8, 9]. Like the structure of Gene Ontology [10] and Disease Ontology, the structure of MeSH as a directed acyclic graph [11] allows the comparison of semantic similarity between two MeSH terms in the graph.

Several MeSH tools have been developed to realize bio-NE recognition, normalization, or comparison. As a MeSH tool for bio-NE recognition and normalization, NLM MeSH has provided an online browser (https://meshb.nlm.nih.gov/search) to parse MeSH terms from the input phrases. However, the browser is neither tolerant to even subtle difference of input phrases from MeSH terms, nor applicable to batch processing. Although some Bio-NE tools based on machine learning method have come out with good performance on specific corporas, they were designed for recognizing certain categories, like diseases and chemicals, of MeSH terms from literature abstracts, and have unknown performance for other categories of MeSH terms or from short biomedical phrases. As MeSH tools for bio-NE comparison, *meshes* [12] and *meshSim* [13] have recently been developed to measure MeSH semantic similarity by using the R dataset MeSH.Hsa.eg.db [3] as data framework. However, the lack of SCRs in MeSH dataset limits the use of tools both *meshes* [12] and *meshSim* for comparing rare diseases such as “alzheimer’s disease 7” and “Bardet-Biedl syndrome 11”. Furthermore, there is still a lack of an integrated one-stop MeSH toolkit to realize bio-NE recognition, normalization, and comparison.

To solve above problems, an integrative python package pyMeSHSim was developed to realize bio-NE recognition, normalization and comparison for MeSH terms. It can directly parse MeSH terms from free biomedical texts and measure the semantic similarity between the MeSH term pairs. Additionally, a lightweight comprehensive MeSH dataset was generated and embedded as the data framework into pyMeSHSim, which enables batch processing and the application of pyMeSHSim to both common diseases and rare diseases.

## Material and methods

### Dataset construction

A comprehensive MeSH dataset is fundamental to MeSH tools. However, the MeSH dataset used by most popular MeSH tools contains only MeSH Main Headings (MHs), a component of MeSH descriptor records, but it contains no SCRs. To construct a comprehensive MeSH dataset, we extracted MeSH information, including MHs, SCRs, and their relations, from Unified Medical Language System (UMLS, 2018AA version) which is a large biomedical thesaurus integrating nearly 200 vocabularies including MeSH [14].

The multiple-to-one relationship between MeSH-synonymous UMLS concepts and MeSH MHs was curated from the table MRSAT in UMLS. For example, the MeSH MH “Alzheimer Disease” (D000544) includes seven MeSH concepts, each of which corresponds to several MeSH entry terms and a UMLS concept (Supplementary Table 1). In our dataset, we included the MeSH MHs and related UMLS concepts, while we excluded the MeSH concept and MeSH entry term information. Moreover, we curated the most useful “parent” and “child” relationship between MeSH MHs from the table MRREL in UMLS.

The one-to-one relationship between MeSH-synonymous UMLS concepts and SCRs was curated from the table MRSAT in UMLS. In our dataset, we included the SCRs and its corresponding UMLS concepts, as well as the “narrower” and “broader” relationship between SCRs and MeSH MHs curated from the table MRREL in UMLS.

The qualifier records and other MeSH descriptor records except MeSH MHs were not included in our dataset. In the study, we used “MeSH term” to refer to MeSH MH or SCR.

### Bio-NE recognition and normalization

The bio-NE recognition were realized by MetaMap [15], a widely used biomedical natural language processing software recognizing UMLS concepts from free texts. Although machine learning methods might have better performance than MetaMap in recommending MeSH MHs to MEDLINE citations, their use were constrained by the requirement of large amount of training data to establish the model and by the potential imbalance of the training data [16]. However, disease phenotypes from GWASdb [17], OMIM [18], and GAD [19] and drug indications in public databases DrugBank [20] and TTD [21] could not provide large amount of training data required by machine learning, while MetaMap required no training data, which was the advantage of MetaMap. The UMLS concepts curated by MetaMap were then converted to MeSH terms based on our dataset. MeSH-synonymous UMLS concepts were directly converted to MHs or SCRs, while non-MeSH-synonymous UMLS concepts, as free texts, were first processed into MeSH-synonymous UMLS concepts and then converted to MHs or SCRs.

### Bio-NE comparison

We compared the bio-NEs based on the similarity between their corresponding MeSH terms. The semantic similarity was usually calculated by graph-based or information content (IC)-based method. The graph-based method measured the node distance between two MeSH terms in the MeSH hierarchical structure, while the IC-based method depended on the specificity and informativeness of MeSH terms [22].

We retrieved the number of publications indexed by MeSH terms using the NCBI E-Utility [23], and calculated the IC values as below.

1 $$ D(d)=\left\{ Descendants\ of\ d\right\} $$

2 $$ P(d)=\frac{freq\left(D(d)\right)}{N} $$

3 $$ IC(d)=-\mathit{\log}\left(P(d)\right) $$

Where *D(d)* is the sum of all the descendent terms of MeSH term *d*; *freq(x)* is the number of publications indexed by term *x*; *N* is the total number of publications indexed by MeSH; and *IC(d)* is the IC value of term *d*.

We implemented the following four IC-based algorithms:

4 $$ {Sim}_{res}\left({d}_1,{d}_2\right)= IC\left( MICA\left\{{d}_1,{d}_2\right\}\right) $$

5 $$ {Sim}_{lin}\left({d}_1,{d}_2\right)=\frac{2\times IC\left( MICA\left\{{d}_1,{d}_2\right\}\right)}{IC\left({d}_1\right)+ IC\left({d}_2\right)} $$

6 $$ {Sim}_{JC}\left({d}_1,{d}_2\right)=1-\mathit{\min}\left(1, IC\left({d}_1\right)+ IC\left({d}_2\right)-2\times IC\left( MICA\left\{{d}_1,{d}_2\right\}\right)\right) $$

7 $$ {Sim}_{rel}\left({d}_1,{d}_2\right)={Sim}_{lin}\left({d}_1,{d}_2\right)\times \left(1-{10}^{- IC\left( MICA\left\{{d}_1,{d}_2\right\}\right)}\right) $$

Where *d*_1_ and *d*_2_ are MeSH terms; *Sim*_*lin*_, *Sim*_*res*_, *Sim*_*rel*_, and *Sim*_*JC*_ correspond to Lin’s [24], Resnik’s [25], Schlicker’s [26], and Jiang and Conrath’s [27] algorithms, respectively; MICA (the most informative common ancestor) is the ancestor of the selected two MeSH terms with the maximal IC value among all ancestors. We designated MICA as 0, which was between MeSH terms from different categories denoted by the first character of the tree number of MeSH terms. For example, MICA between the MeSH terms “Tauopathies” (tree number: “C10.574.945”) and “Schizophrenia” (tree number: “F03.700.750”) is 0 because they belonged to different categories (“C” for diseases vs “F” for psychiatry and psychology).

We also implemented the graph-based Wang’s [28] algorithm as below.

8 $$ A(d)=\left\{ Ancestor\ of\ d\right\} $$

9 $$ {S}_d(a)=\mathit{\max}\left\{{\omega}^{n_a}\right\},a\in A(d) $$

10 $$ {SV}_d=\sum \limits_{t\in A(d)}{S}_d(t) $$

11 $$ {Sim}_{Wang}\left({d}_1,{d}_2\right)=\frac{\sum \limits_{t\in A\left({d}_1\right)\cap A\left({d}_2\right)}\left(\ {S}_{d_1}(t)+{S}_{d_2}(t)\right)\ }{SV_{d_1}+{SV}_{d_2}} $$

Where *d* is a MeSH term; *A(d)* is the ancestors deduced from tree numbers of *d*; *n*_*a*_ is the number of edges between *d* to *a*; *S*_*d*_(*a*) is the semantic contribution of *a* to *d*; *SV*_*d*_ is the total semantic contributions of all ancestors to *d*; *Sim*_*Wang*_(*d*_1_, *d*_2_) is Wang’s algorithm score between MeSH terms *d*_1_ and *d*_2_; *ω* is a tuneable weight in [0,1] range used to measure the relation between two terms. In this study, we tuned *ω* from 0 to 1 with a step of 0.1 to test the robustness of our results (Supplementary Table 2, Supplementary figure 1A, 1B), and set it to 0.6, when pyMeSHSim using Wang’s algorithm had the highest correlation with meshes for all the algorithms.

Noteworthily, both IC-based and graph-based methods depended on the tree number, but some MeSH terms may have more than one tree number, thus resulting in multiple similarity values between one pair of MeSH terms. We retained only the maximal similarity value between two MeSH terms.

### Package detail

**Fig. 1:**
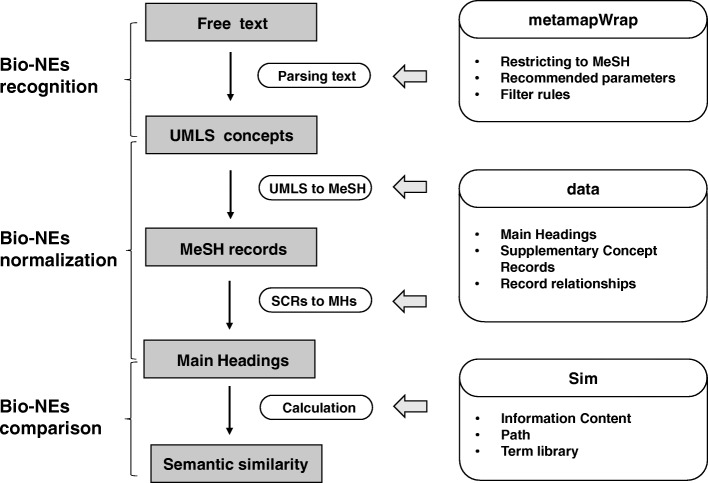
The components and workflow of pyMeSHSim. pyMeSHSim consists of three subpackages, including *metamapWrap*, *data* and *Sim*. In bio-NE recognition, *metamapWarp* curates the UMLS concepts from free text. In bio-NE normalization, *data* translates UMLS concepts to MeSH terms, and maps SCRs to MHs using selected records and relationships between records in MeSH. In bio-NEs comparison, *Sim* uses IC-based and graph-based methods to measure semantic similarity between two bio-NEs

The pyMeSHSim consists of three subpackages (1) the *metamapWrap* subpackage recognizing bio-NEs from the text, (2) the *data* subpackage normalizing UMLS concepts into MeSH terms by the embedded MeSH dataset, and (3) the *Sim* subpackage comparing semantics of MeSH terms by measuring the distance between MeSH terms (Fig. 1). Detailed descriptions of the subpackages and their parameters are provided in the reference manual (Supplementary File 1, https://pymeshsim.readthedocs.io/en/latest/).

1. The metamapWrap subpackage

The bio-NE recognition and normalization of pyMeSHSim were realized by the *metamapWrap* subpackage which was a wrapper for MetaMap [15]. The subpackage *metamapWrap* curated MeSH-synonymous UMLS concepts from free texts including non-MeSH-synonymous UMLS concepts, and then converted the curated MeSH-synonymous UMLS concepts into corresponding MeSH terms via the data subpackage. We set parameters “-N -J semantic_type _list -R MSH -I -z -conj -Q 4 -silent --sldi”, where semantic_type list was the list of disease-related semantic types (corresponding to “inpo,dsyn,phpranab,orgf,clna,hlca,genf,orga,neop,emod,inbe,lbtr,anst,npop,celc,cell,bpoc,acty,mobd,celf,evnt,sosy,patf,tisu,moft,fndg,bdsu,ortf,menp,acab,comd,sbst,cgab”, as can be seen in the manuals) as the default of pyMeSHSim. Users can customize the parameters to suit their needs.

2) The data subpackage

The MeSH dataset was embedded into the *data* subpackage in bcolz format with a corresponding data interface (Supplementary Table 3). It included five tables: (1) Table MainHeadingDetailData contained all the MH information, including MeSH unique id, tree code, prefer name, category, term semantic type, IC frequency, and UMLS id. The semantic type was derived from the UMLS table MRSTY, and each UMLS concept was characterized by at least one of the 133 semantic types [29]; (2) Table SupplementMainHeading contained all the UMLS concepts related to MHs; (3) Table RNDetailData stored the basic information of SCRs; (4) Table RNandRBRel exhibited the narrower-and-broader relationship between SCRs and MHs; (5) Table ParentChildRel contained the fundamental tree structure. The five tables made possible the conversion of UMLS concepts into MeSH terms and the measurement of the semantic similarity between MeSH terms.

3) The Sim subpackage

The bio-NE comparison of pyMeSHSim was conducted with the *Sim* subpackage by measuring the distance between MeSH terms. Each narrower record of the SCR was converted into one or more broader terms of MHs before the measurement. Like the tool *meshes*, pyMeSHSim offered five representative semantic similarity measurements, including four information content (IC) based (Lin’s, Resnik’s, Schlicker’s, and Jiang and Conrath’s) and one graph-based (Wang’s) algorithms.

## Results

### Evaluation with OMIM phenotypes

**Fig. 2:**
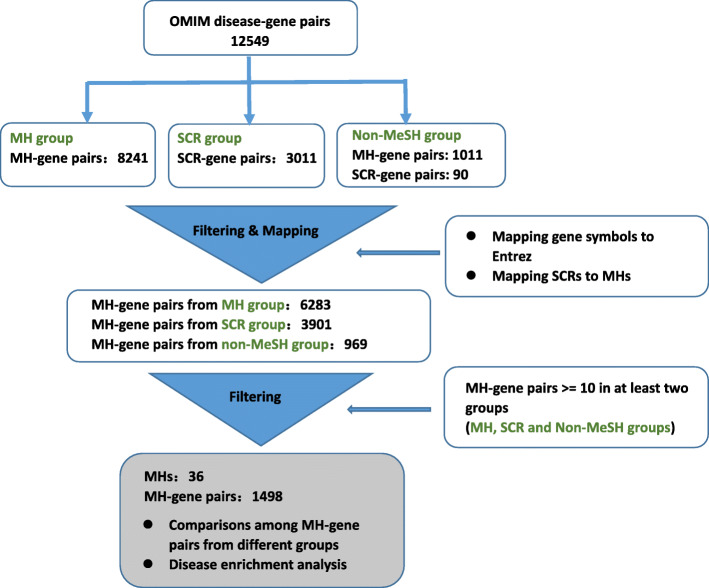
OMIM UMLS diseases processing pipeline. MeSH-synonymous UMLS concepts were mapped to MHs or SCRs by pyMeSHSim directly. Meanwhile, non-MeSH-synonymous UMLS concepts were processed as free texts into MeSH-synonymous UMLS concepts, and then mapped to MeSH terms. All gene symbols were mapped to Entrez IDs. SCRs were mapped to its broader MHs. MHs with at least 10 genes in at least two groups were remained for further analysis

**Fig. 3:**
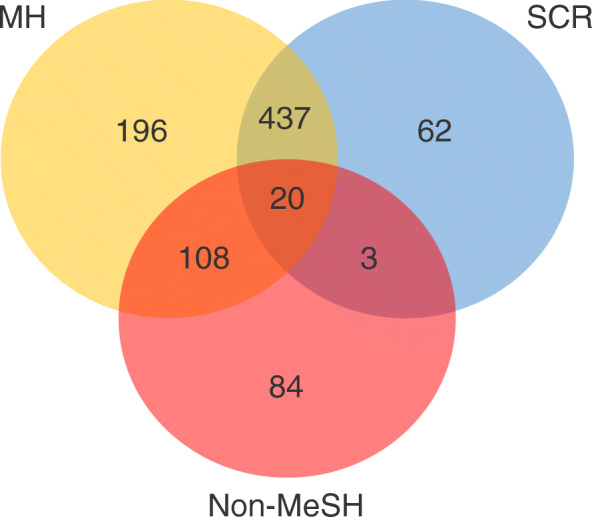
Venn diagrams. Venn diagram of MH-gene pairs in MH, SCR and Non-MeSH groups. Yellow, red and blue circles represent MH, Non-MeSH and SCR groups respectively. The digital shows number of MH-gene pairs in each group and overlapped number of MH-gene pairs between different groups

To test whether the introduction of SCRs and our curation strategy of non-MeSH-synonymous UMLS concepts contributes to improving the performance of pyMeSHSim in bio-NE recognition, we compared the genes annotated with MeSH MHs and SCRs from OMIM [18] phenotype-gene pairs. The OMIM phenotype-gene pairs were collected from the database disease-connect [30], which used MetaMap to process the disease phenotypes into MeSH-synonymous and non-MeSH-synonymous UMLS concepts. MeSH-synonymous UMLS concepts were directly converted into MHs and SCRs by using pyMeSHSim. Subsequently, SCRs were further converted into their “broader” MHs. Non-MeSH-synonymous UMLS concepts, as free texts, were processed into MeSH-synonymous UMLS concepts. Based on the source of their corresponding UMLS concepts, we classified OMIM phenotypes into MH, SCR, and non-MeSH groups. And then, we compared the genes corresponding to the same MHs from all the three groups (Fig. 2). The genes without Entrez IDs were excluded, since Entrez IDs were required for the following disease enrichment analysis. The MHs with less than 10 genes in at least two groups were also excluded. After the filtering, 36 MHs and 1498 MH-gene pairs (Supplementary Table 4) were remained, including 761 MH-gene pairs from MH group, 522 from SCR group, and 215 from non-MeSH group. About 87.5% MH-gene pairs in SCR group were also present in MH group, indicating high overlap of genetic features between subtype diseases and its corresponding MH diseases, and validating the significance of SCRs in disease curation (Fig. 3). Additionally, the 59.5% overlap of MH-gene pairs was found between non-MeSH group and MH group and 10.7% overlap between non-MeSH group and SCR group, indicating the effectiveness of our curation strategy of non-MeSH-synonymous UMLS concepts.

To further validate the reasonability of introducing SCR and our curation strategy of non-MeSH-synonymous UMLS concepts, we hypothesized that the additional MH-gene pairs derived from SCRs and non-MeSH-synonymous UMLS concepts should improve the gene enrichment in the MH diseases. We remained the seven MHs with at least 5 non-overlap MH-gene pairs in SCR group and non-MeSH group, and tested the enrichment of genes corresponding to MHs in the diseases by using the UMLS-based disease enrichment analysis tool DOSE [31]. For each of the seven MHs, the addition of genes from SCR and non-MeSH groups led to more significant enrichment in the disease mapped to the MH (Table 1). Especially, the addition of 50 genes of the MeSH MH Osteochondrodysplasias (D010009) from SCR and non-MeSH groups to the 14 genes from the MH group led to the higher *p* value (6.57E-35 vs 8.87E-19) of enrichment in the disease Osteochondrodysplasias (Table 1), suggesting the contribution of the introduced SCRs and curation strategy of non-MeSH-synonymous UMLS concepts to the improved performance of pyMeSHSim in bio-NE recognition and normalization.

**Table 1:** Disease enrichment analysis of the genes assigned to the MHs before and after addition of MH-gene pairs from SCR and non-MeSH groups

OMIM diseases^**1**^		MH-gene pairs (MH group / all)^**2**^	Enriched UMLS diseases with DOSE			***P*** value (MH group / all)^**4**^
MH ID	MH description		UMLS ID	UMLS description	MH ID^**3**^	
D057130	Leber Congenital Amaurosis	17/22	C0339527	Leber Congenital Amaurosis	D057130	3.43E-33 / 1.45E-42
D020754	Spinocerebellar Ataxias	23/28	C0087012	Ataxia, Spinocerebellar	D020754	1.93E-30 / 2.84E-38
D052177	Kidney Diseases, Cystic	19/25	C1691228	Cystic Kidney Diseases	D052177	8.05E-19 / 2.37E-20
D010009	Osteochondrodysplasias	14/64	C0029422	Osteochondrodysplasias	D010009	8.87E-19 / 6.57E-35
D002925	Ciliary Motility Disorders	26/31	C0008780	Ciliary Motility Disorders	D002925	1.60E-23 / 3.90E-33
D015419	Spastic Paraplegia, Hereditary	28/36	C0037773	Spastic Paraplegia, Hereditary	D015419	1.22E-37 / 2.71E-45
D007938	Leukemia	18/51	C0085669	Acute leukemia	D007938	3.26E-10 / 6.63E-26

^1^ The OMIM diseases were collected from the database disease-connect (34) with at least five MH-gene pairs outside the MH group.

^2^ (Number of MH-gene pairs in MH group) / (number of MH-gene pairs in all the three groups including MH, SCR and non-MeSH group).

^3^ The MH ID was mapped from the UMLS ID by pyMeSHSim.

^4^ (The enrichment *P* value of genes in MH group) / (The enrichment *P* value of genes in all the three groups).

### Evaluation with GWAS phenotypes

To evaluate the performance of pyMeSHSim on bio-NE recognition, we took the manual work of Nelson’s group in parsing 461 GWAS phenotypes to MeSH terms as the gold standard, and compared the performance of pyMeSHSim with DNorm and TaggerOne, which are the state-of-the-art machine learning based tools for locating and identifying disease and chemical concepts [32–34].

**Fig. 4:**
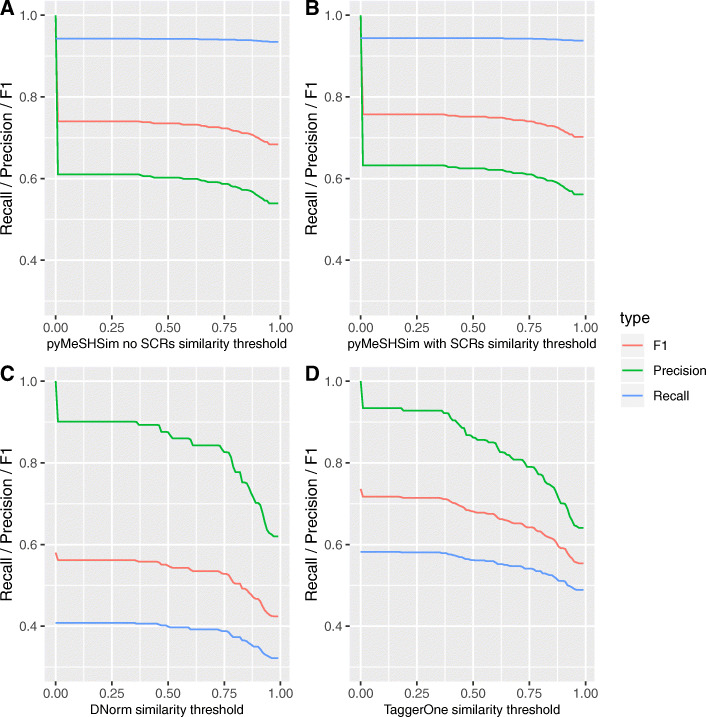
Recall, Precision and F1 of pyMeSHSim, DNorm and TaggerOne. **a**-**d**. Performance of pyMeSHSim without SCRs (**a**), pyMeSHSim with SCRs (**b**), DNorm (**c**) and TaggerOne (**d**). The similarity between MeSH terms identified by the tools and Nelson’s manual work were called as a true positive or false positive when their similarity was higher or lower than the determined threshold. When the similarity threshold is set to 1, only perfect matched terms would be considered as true positives. The recall ($\frac{TP}{TP+ FN}$), precision ($\frac{TP}{TP+ FP}$) and F1 ($\frac{2\times precision\times recall}{precision+ recall}$) of the tools were calculated at each similarity threshold

DNorm and TaggerOne integrated different Lexical resources as training data, and could recognize MeSH terms and OMIM terms from free text. In the performance comparison, we only extracted the MeSH results from these two softwares. PyMeSHSim successfully recognized MeSH terms from 442 (96%) GWAS phenotypes, while DNorm and TaggerOne only identified 129 (28%) and 192 (42%) (Supplementary Table 5). There were 158 phenotypes specifically identified by pyMeSHSim but not by DNorm/TaggerOne. Regarding the categories of recognized MeSH terms, pyMeSHSim successfully identified terms in 15/17 categories, while DNorm and TaggerOne, which were designed for disease or chemical entity recognition, identified terms mainly in “C” (Diseases) and “F” (Psychiatry and Psychology) categories (Supplementary Table 6). Even for phenotypes in the “C” category, pyMeSHSim (> 0.94) showed higher recall than DNorm (> 0.32) and TaggerOne (> 0.49) across all the similarity thresholds used to determine matches with Nelson’s manual work as true positives (Supplementary Table 5, Fig. 4). Despite the lower precision of pyMeSHSim (> 0.56) than DNorm (> 0.62) and TaggerOne (> 0.64), the differences in precision were subtle when consider only perfect match (Table 2, Fig. 4), and the overall performance F1 of pyMeSHSim (> 0.70) was always higher than DNorm (> 0.42) and TaggerOne (> 0.55) (Fig. 4). The lower performance of DNorm and TaggerOne maybe since they were not MeSH term taggers. Additionally, the recall, precision and F1 were all higher for pyMeSHSim with SCRs than that without SCRs, demonstrating the contribution of SCRs to improved performance of pyMeSHSim in bio-NE recognition and normalization.

**Table 2:** Performance comparing pyMeSHSim, DNorm, TaggerOne to Nelson’s manual work with similarity threshold set to 1

Method	Recall^a^	Precision^b^	F1^c^
**pyMeSHSim (with SCRs)**	0.94	0.56	0.70
**pyMeSHSim (no SCRs)**	0.94	0.54	0.68
**DNorm**	0.32	0.62	0.42
**TaggerOne**	0.49	0.64	0.55

^a^$all=\frac{TP}{TP+ FN}$, where TP (true positive) is the number of phenotypes whose parsing results matched the manual work at determined similarity threshold. The similarity between MeSH terms identified by the two methods were measured with Lin score, and called as a TP or FP when their similarity was higher or lower than the determined threshold. FN (false negative) is the number of unrecognized phenotypes.

^b^$cision=\frac{TP}{TP+ FP}$, where FP is the number of phenotypes whose parsing results mismatched the manual work at determined similarity threshold.

^c^$$ 1=\frac{2\times precision\times recall}{precision+ recall} $$ .

We then investigated the phenotypes in the “C” category specifically tagged by pyMeSHSim or DNorm/TaggerOne with the same MeSH term as Nelson’s manual work, and found 38 phenotypes specifically identified by pyMeSHSim (Supplementary Table 7), while only five by DNorm/TaggerOne (Supplementary Table 8). The 38 phenotypes specifically identified by pyMeSHSim included 26 phenotypes tagged with related MeSH terms by DNorm/TaggerOne (similarity Lin score > 0), and 12 missed by them. Among the 12 phenotypes, “Graves` disease” (D006111), “Paget’s disease” (D010001), and “Behcet’s disease” (D001528) might be missed due to special symbol “`”. Meanwhile, the five phenotypes not perfectly identified by pyMeSHSim included three tagged with related MeSH terms by pyMeSHSim, and two missed by it (“Tumor biomarkers” and “Coronary artery calcification”). The phenotype “Tumor biomarkers” was correctly recognized by pyMeSHSim as D014408 (Tumor biomarkers), while tagged as D009369 (Neoplasms) by Nelson’s group and DNorm. The other phenotype “Coronary artery calcification” was mistakenly identified as D002113 (Calcification, Physiologic) by pyMeSHSim, while as D061205 (Vascular Calcification) by Nelson and TaggerOne. These results of error analysis demonstrated better performance of pyMeSHSim than DNorm and TaggerOne in recognizing MeSH terms from short biomedical phrases like GWAS phenotypes.

We further compared the parsing results of pyMeSHSim with Nelson’s manual work, and found 114 phenotypes differently tagged (similarity Lin score = 0) and 17 missed by pyMeSHSim. The manual work preferred mapping the phenotypes to disease category (C). For example, phenotypes like “Vitamin E levels”, “Hematology traits” and “Pulmonary function” were parsed as “Vitamin E Deficiency” (D014811), “Hematologic Diseases” (D033461) and “Lung Diseases” (D008171) by Nelson’s group, while identified as “Vitamin E” (D014810), “Hematology” (D006405) and “Lung” (D008168) by pyMeSHSim. However, such preference of the manual work could lead to bias. For example, “Eye color”, “Hair color” and “Serum urate” were parsed as “color vision defects”, “hair diseases” and “urinary calculi” by Nelson’s group, while as “Color, Eye”, “Color, Hair” and “Acid, Uric” by pyMeSHSim (Supplementary Table 5). Therefore, at least a part of the parsing differences between the manual work and pyMeSHSim were attributed to human bias in the manual work. Meanwhile, among the 17 phenotypes not recognized by pyMeSHSim, “IgG levels”, “IgM levels”, “IgE levels”, “PR interval” and “QT interval” might be missed due to the abbreviations inside (Supplementary Table 5).

To test the semantic similarity function of pyMeSHSim, we calculated all the semantic similarities between the curated MeSH terms using pyMeSHSim and the latest semantic analysis tool *meshes* (Supplementary Table 2). The similarity calculated by both packages was 1 when the MeSH terms were the same, and was 0 when MeSH terms were of different categories. The 55 GWAS phenotypes with the different term pairs in the same category were found resulting from the recognition respectively via pyMeSHSim and Nelson’s group work. The pyMeSHSim succeeded in calculating the similarities between the term pairs of all the 55 phenotypes, while *meshes was* only capable of comparing MH-MH pairs, and it failed to compare SCR-MH pairs of 15 phenotypes (Supplementary Table 2). Of the 15 SCRs parsed by pyMeSHSim, 13 were mapped to the same MHs as parsed by Nelson’s group. The similarity correlation of the remaining 40 term pairs between pyMeSHSim and *meshes* was 0.89 (Rel’s)-0.97 (Res’) (Table 3, Supplementary Table 2, Supplementary figure 1B), demonstrating similar, if not better, performance of pyMeSHSim to that of *meshes* in bio-NE comparison.

**Table 3:** Correlation of calculated semantic similarities between pyMeSHSim and *meshes*

Method	Lin’s	Res’	Jiang’s	Rel’s	Wang’s
**Correlation coefficient**	0.97	0.99	0.89	0.98	0.97
***P*value**	< 2.2e-16	< 2.2e-16	1.2e-14	< 2.2e-16	< 2.2e-16

## Discussions

### Effectiveness of pyMeSHSim

PyMeSHSim aims to provide users a one-stop MeSH toolkit for bio-NE recognition, normalization and comparison, and multiple efforts were made to confirm its effectiveness. For example, (i) We compared the performance of pyMeSHSim in bio-NE recognition and normalization with manual work in parsing GWAS phenotypes, and found high consistency between them, indicating the great potential of pyMeSHSim for aiding professional manual curation of bio-NEs; (ii) We compared the performance of pyMeSHSim in bio-NE recognition and normalization with another two tools base on machine learning methods, and showed higher sensitivity and accuracy of pyMeSHSim in parsing short biomedical phases like GWAS phenotypes; (iii) We converted the OMIM phenotypes to MeSH terms using pyMeSHSim, and demonstrated improved effectiveness in bio-NE recognition and normalization by including SCRs in its embedded dataset; (iv) We compared the similarity measurement between pyMeSHSim and *meshes* and showed comparable performance in bio-NE comparison.

### Caveat

Considering that MeSH is one of the most widely used biomedical vocabulary, pyMeSHSim will further contribute to data integration. In addition, the introduction of SCRs to the implemented dataset enables pyMeSHSim to handle rare diseases in public databases like OMIM and Orphanet (www.orpha.net). However, whether general concepts such as MHs or specific concepts such as SCRs are preferable will depend on the end use. Users should be cautious to select the according right terms in using pyMeSHSim.

## Conclusions

We developed pyMeSHSim, an integrative, lightweight, and data-rich python package for biomedical text mining. To the best of our knowledge, this is the first one-stop MeSH toolkit integrating the functions of bio-NE recognition, normalization and comparison. PyMeSHSim is expected to be widely used as a powerful tool in bioinformatics, computational biology, and biomedical research.

## Supplementary information

**Additional file 1 **: **Supplementary figure 1.** Determination of the parameter weight ω for Wang’s algorithm based on the semantic similarity between the 40 MeSH term pairs in the evaluation with GWAS phenotypes. A. Violin plot of the semantic similarity calculated by pyMeSHSim with Jiang and Conrath’s (jiang), Lin’s (lin), Resnik’s (res), Schlicker’s (rel), and) and Wang’s (wang) algorithms. The effect of weight ω for Wang’s algorithm was test by tuning it from 0 to 1 (weight_0.0 to weight_1.0). B. The Pearson’s correlation between the results of pyMeSHSim (Y axis) and meshes (X axis). The 40 MeSH pairs with semantic similarity between 0 ~ 1 were shown in Supplementary Table 2. The weight ω was set to be 0.6 when pyMeSHSim had the highest correlation with meshes for all the algorithms.

**Additional file 2.**

**Additional file 3 Supplementary Table 1.** MeSH terms and UMLS concepts correspond to MeSH MH D000544.

**Additional file 4 Supplementary Table 2.** GWAS phenotypes parsed by Nelson’s group and pyMeSHSim, and the semantic similarity between them calculated by pyMeSHSim and meshes.

**Additional file 5 Supplementary Table 3.** Number of MHs and SCRs in each MeSH category.

**Additional file 6 Supplementary Table 4**. The OMIM MH-gene pairs from MH, SCR, and Non-MesH.groups.

**Additional file 7 Supplementary Table 5**. GWAS phenotypes parsed by Nelson’s group and pyMeSHSim, TaggerOne and DNorm. the semantic similarity between them calculated by pyMeSHSim. pyMeSHSim_Score is semantic similarity between Nelson_MeSH _ID and pyMeSHSim_MeSH_ID, taggerOne_score is semantic similarity between Nelson_MeSH _ID and TaggerOne_MeSH_ID, DNorm_score is semantic similarity between Nelson_MeSH _ID and Dnorm_MeSH_ID.

**Additional file 8 Supplementary Table 6.** MeSH term number in each category correctly identified by pyMeSHSim, Dnorm, TaggerOne and Nelson’s manual work.

**Additional file 9 Supplementary Table 7.** pyMeSHSim perfectly recognized MeSH term, but DNorm and TaggerOne failed. The semantic similarity between them calculated by pyMeSHSim. pyMeSHSim_Score is semantic similarity between Nelson_MeSH _ID and pyMeSHSim_MeSH_ID, taggerOne_score is semantic similarity between Nelson_MeSH _ID and TaggerOne_MeSH_ID, DNorm_score is semantic similarity between Nelson_MeSH _ID and Dnorm_MeSH_ID.

**Additional file 10 **: **Supplementary Table 8**. DNorm or TaggerOne perfectly recognized MeSH terms, but pyMeSHSim failed. The semantic similarity between them calculated by pyMeSHSim. pyMeSHSim_Score is semantic similarity between Nelson_MeSH _ID and pyMeSHSim_MeSH_ID, taggerOne_score is semantic similarity between Nelson_MeSH _ID and TaggerOne_MeSH_ID, DNorm_score is semantic similarity between Nelson_MeSH _ID and Dnorm_MeSH_ID.

## Data Availability

All data generated or analysed during this study are included in this published article and its supplementary information files.

## Abbreviations

bio-NE: Biomedical named entity
UMLS: Unified Medical Language System
MeSH: Medical Subject Heading
MH: Main headings
SCR: Supplementary concept record
IC: Information content
MICA: The most informative common ancestor
GWAS: Genome wide association studies
OMIM: Online Mendelian Inheritance in Man
