# Supplementary figures and images for: pyMeSHSim: an integrative python package for biomedical named entity recognition, normalization, and comparison of MeSH terms
Source: BMC Bioinformatics. 2020 Jun 18;21:252. doi: 10.1186/s12859-020-03583-6 (PMC7301509; doi:10.1186/s12859-020-03583-6)

A.

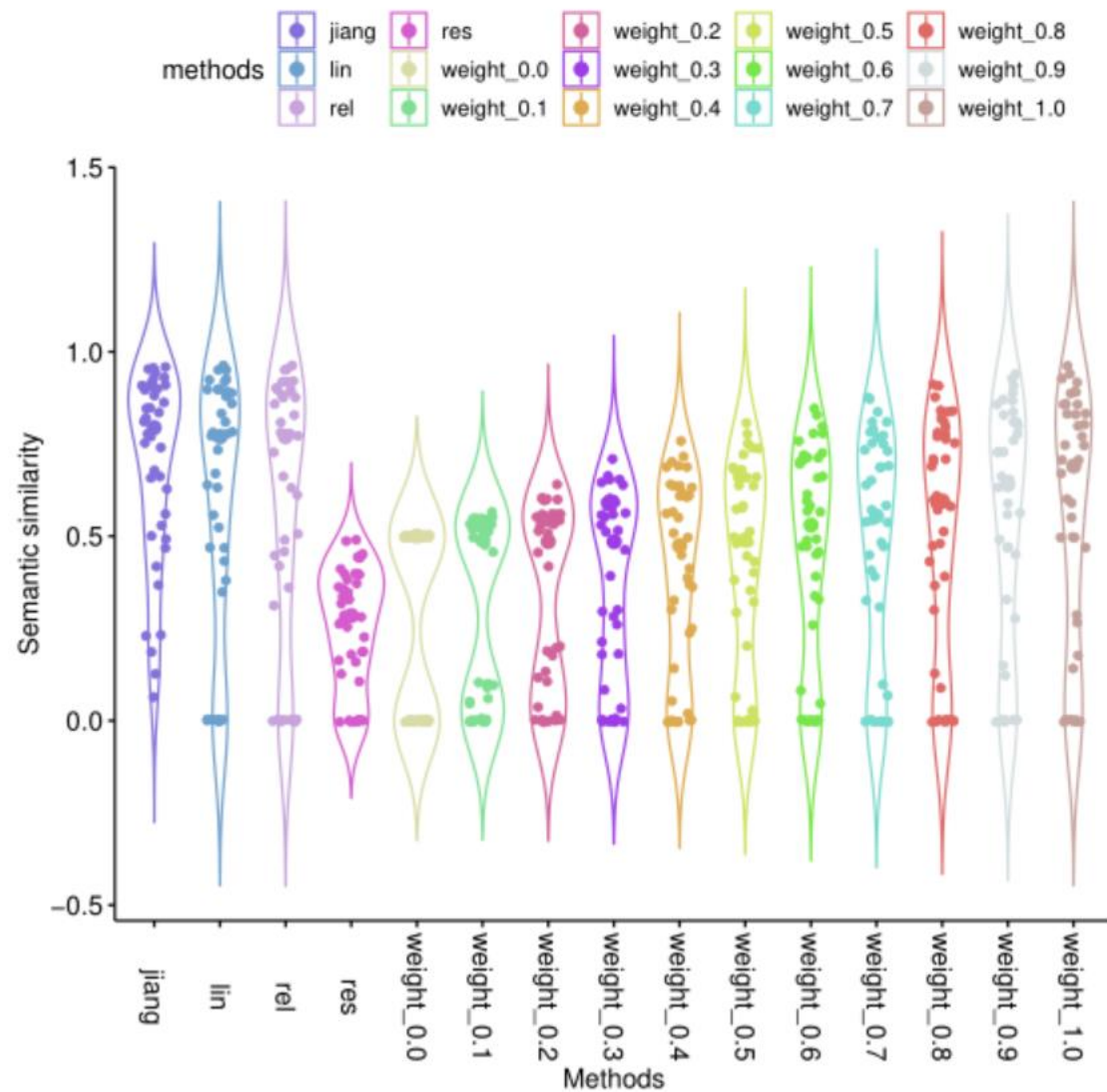

B.

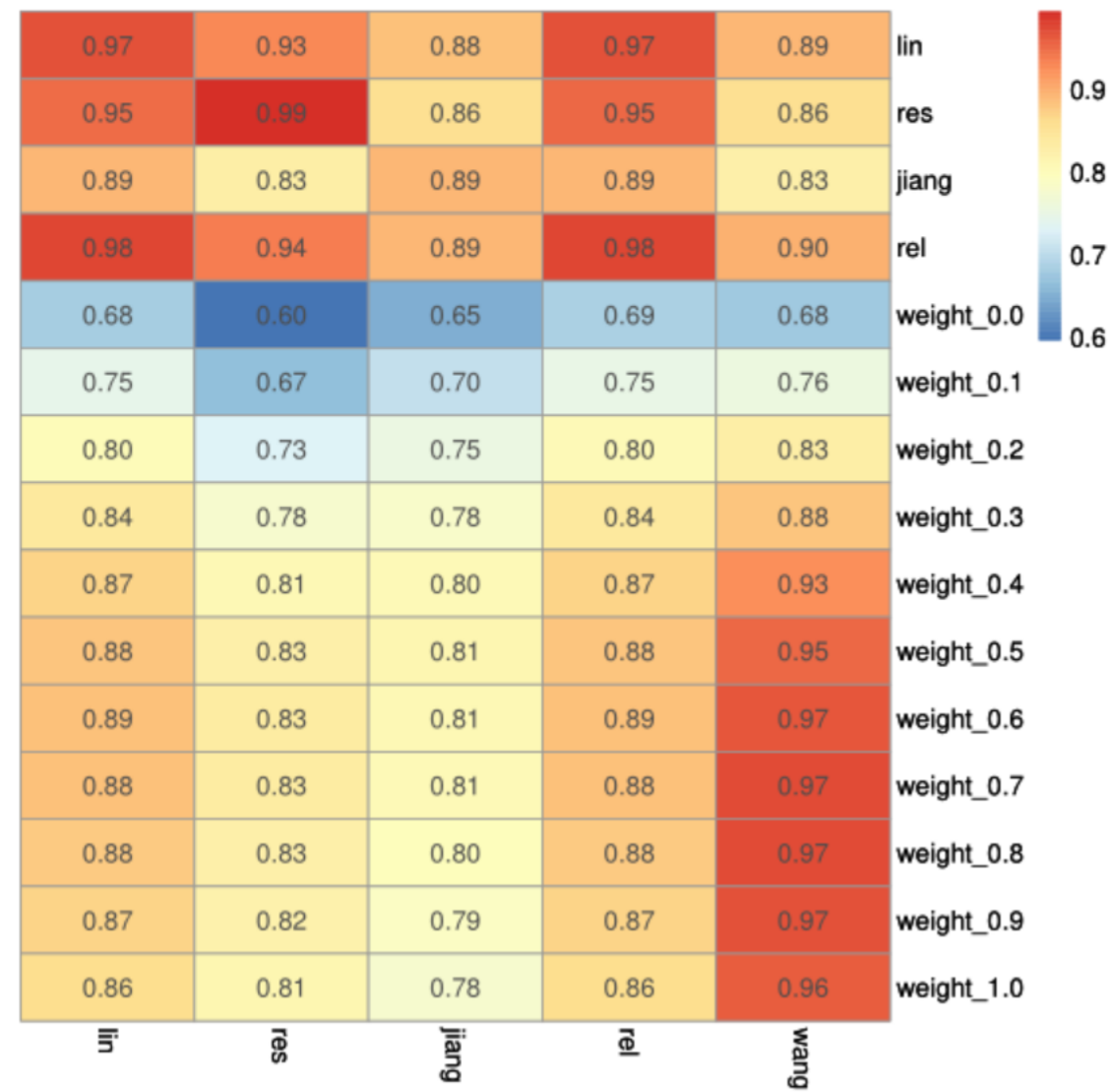

Supplement: Supplementary file 1 — Additional file 1 : Supplementary figure 1. Determination of the parameter weight ω for Wang’s algorithm based on the semantic similarity between the 40 MeSH term pairs in the evaluation with GWAS phenotypes. A. Violin plot of the semantic similarity calculated by pyMeSHSim with Jiang and Conrath’s (jiang), Lin’s (lin), Resnik’s (res), Schlicker’s (rel), and) and Wang’s (wang) algorithms. The effect of weight ω for Wang’s algorithm was test by tuning it from 0 to 1 (weight_0.0 to weight_1.0). B. The Pearson’s correlation between the results of pyMeSHSim (Y axis) and meshes (X axis). The 40 MeSH pairs with semantic similarity between 0 ~ 1 were shown in Supplementary Table 2. The weight ω was set to be 0.6 when pyMeSHSim had the highest correlation with meshes for all the algorithms. [file 12859_2020_3583_MOESM1_ESM.pdf]
