# Supplementary material for: pyMeSHSim: an integrative python package for biomedical named entity recognition, normalization, and comparison of MeSH terms
Source: BMC Bioinformatics. 2020 Jun 18;21:252. doi: 10.1186/s12859-020-03583-6 (PMC7301509; doi:10.1186/s12859-020-03583-6)
Supplement: Supplementary file 2 — Additional file 2. [file 12859_2020_3583_MOESM2_ESM.pdf]

pyMeSHSim Documentation

Release 0.0.1

Zhihui Luo

Feb 14, 2020

Contents:

1 Introduction **1**

1.1 pyMeSHSim at glance 1

1.1.1 Biomedical named entity (Bio-NE) recognition, normalization, and comparison 1

1.1.2 MeSH 1

1.1.3 pyMeSHSim 1

1.1.4 Currently, pyMeSHSim consists of three subpackages: 2

1.1.5 Download 2

2 Installation **3**

2.1 Requirements 3

2.2 MetaMap installation 3

2.3 Installation of pandas and bcolz 4

2.4 Installation of pyMeSHSim 4

3 Tutorial **5**

3.1 Important parameters introduction 5

3.1.1 MetamapWrap 5

3.1.2 Data 7

3.1.3 Sim 8

3.2 pipeline script 8

3.3 Parsing free text 9

3.3.1 Parsing short sentence 9

3.3.2 Parsing long sentence 10

3.3.3 Filtering the result 11

3.4 Term library 13

3.4.1 Obtaining MeSH terms from UMLS concepts 13

3.4.2 Obtaining MeSH term detail from MeSH ID 13

3.4.3 Obtaining UMLS concept from MeSH ID 14

3.4.4 Obtaining the category of MeSH terms 14

3.4.5 Obtaining narrow or broad terms of a mesh term 14

3.4.6 Obtaining parent or child terms of a MeSH term 15

3.4.7 Obtaining the top term of a MeSH term 15

3.4.8 Obtaining the ancestors or offsprings 15

3.4.9 Retrieving MeSH ID by MeSH tree code 15

3.4.10 Obtaining the preffered name by MeSH ID 15

3.5 Calculating similarity 16

3.5.1 Calculating similarity between MeSH terms 16

4 Library reference 17
4.1 data 17
4.1.1 pyMeSHSim.data.createData 17
4.1.2 pyMeSHSim.data.dataInterface 17
4.1.3 pyMeSHSim.data.duiFunc 17
4.2 metamapWrap 17
4.2.1 pyMeSHSim.metamapWrap.Concept 17
4.2.2 pyMeSHSim.metamapWrap.MetamapInterface 17
4.3 Sim 17
4.3.1 pyMeSHSim.Sim.ICScore 18
4.3.2 pyMeSHSim.Sim.PathScore 18
4.3.3 pyMeSHSim.Sim.MeSHProcess 18
4.3.4 pyMeSHSim.Sim.similarity 18

5 Indices and tables 19

CHAPTER 1

Introduction

1.1 pyMeSHSim at glance

1.1.1 Biomedical named entity (Bio-NE) recognition, normalization, and comparison

The recognition and normalization of bio-NE, especially for diseases, play an important role in clinical and biomedical research, such as clinical decision support, cohort identification, pharmacovigilance, and drug repositioning. For example, bio-NE recognition and normalization are prerequisites for semantic analysis, including semantic comparison of bio-NEs in drug repositioning. However, there are multiple synonyms, abbreviations and variations for bio-NEs, making it challenging to curate bio-NEs from free biomedical text or clinical narrative text.

1.1.2 MeSH

We extracted bio-NEs from free biomedical text and measured semantic similarity between the bio-NEs based on the Medical Subject Headings(MeSH).

MeSH is a medical vocabulary resource curated by the National Library of Medicine (NLM). It provides a hierarchically-organized terminology for indexing and cataloging of biomedical information in MEDLINE/PubMed and other NLM databases. Moreover, MeSH is organized as a directed acyclic graph, laying the foundation for computing semantic similarities between two concepts.

Although MeSH has potential for bio-NE recognition, normalization, and comparison , there is still a lack of MeSH tools to automatically recognize bio-NEs from free text and measure the semantic similarity between bio-NEs after normalization.

1.1.3 pyMeSHSim

Here, we developed an integrative, lightweight and data-rich python package named pyMeSHSim to curate MeSH terms from free text and measure the semantic similarity between the MeSH terms.

1.1.4 Currently, pyMeSHSim consists of three subpackages:

- **data subpackage**
  - The data subpackage has reorganized the MeSH information in bcolz format.
  - It is lightweight and data-rich.
  - It contained the main heading concepts, unique DescriptorUI, MeSH Tree code, and correspond UMLS ID.
  - It contained all narrow concepts of the main heading concepts. It reserved the parent-child relationships and RN/RB relationships for all concepts.
- **metamapWrap subpackage**
  - It provided some filter rules for parsing the free text.
  - It provided a unified interface to create the MeSH concept objects.
- **Sim subpackage**
  - It provided useful APIs to retrieve the MeSH dataset.
  - It implemented four methods of semantic similarity measures based on information content.It implemented one method of semantic similarity measures based on path.

More details can be seen in the reference.

1.1.5 Download

This package can be download at github repository pyMeSHSim.

CHAPTER 2

Installation

2.1 Requirements

- Software
  - MetaMap 2016v2
- Python packages
  - python 3.6
  - pandas
  - bcolz>=1.2.1

2.2 MetaMap installation

MetaMap is the base implement of the subpackage metamapWrap.
You need to activate a UMLS Terminology Services (UTS) account to fetch MetaMap. Please see MetaMap for more information.

MetaMap depends on Java. To install openJDK:

```
$ sudo apt install default-jdk
```

After downloading MetaMap and Extracting it, you can install it by:

```
$ cd ./public_mm/
$ bash ./bin/install.sh
```

At this point, you have successfully installed MetaMap.

Please add the bin directory to the environment variable PATH in bashrc for convenience:

```
$ export PATH=$PATH:/path_to_MetaMap/bin
```

Then, launch MetaMap server before running pyMeSHSim:

```
$ skrmedpostctl start
$ wsdserverctl start
```

2.3 Installation of pandas and bcolz

To install python package pandas and bcolz:

```
$ pip3 install pandas
$ pip3 install bcolz
```

2.4 Installation of pyMeSHSim

To install pyMeSHSim from source code:

```
$ git clone https://github.com/luozhhub/pyMeSHSim.git
$ cd pyMeSHSim
$ python3 ./setup.py install
```

CHAPTER 3

Tutorial

3.1 Important parameters introduction

PyMeSHSim provided three subpackages metamapWrap, Data, and Sim. The Data subpackage was the foundation of metamapWrap and Sim. It contained a redesigned data frame of MeSH terms, including main headings, supplementary concept records, and relations between them. That made MeSH term interpretation more quickly and efficiently. The metamapWrap was the essence of biomedical named entity recognition (bio-NE). It would invoke metamap to parse the free text. Then, metamapWrap converted the result to MeSH terms through the Data subpackage. Besides the above two functions, Sim had implemented five semantic similarity measurement methods. In totally, the functions of pyMeSHSim could be summarized into 4 point:

- Translating free text to MeSH term
- Lightweight data frame for MeSH
- Plenty of MeSH term library
- Five algorithms to measure semantic distance

To make pyMeSHSim easier to be underattended and used. We would illustrate all parameters that may affect the performance of it in this tutotial. In addition, we provided a pipeline script as a batch processing example. Users can refer to the pipeline script or design their own program.

3.1.1 MetamapWrap

MetamapWrap was a wrapper for metamap. metamapWrap combined the functions of metamap and the MeSH vocabulary, then realized bio-NE recognition and normalization. it invoked the metamap to parsed free text to UMLS concepts, then converted the UMLS concepts to MeSH terms via the Data subpackage. Although, the metamap reference had a detail description for its usage, we would introduce some important parameters accompany with pyMeSHSim examples here. The main parameters were listed in extended table 1.

Table 1: important parameters in etamapWrap

Parameters	Values	Description
source	MSH, MTH, OMIM … . Default is MSH	-R in metamap. Vocabulary source. Restricting the metamap to use which thesaurus to parse the free text.
semantic_types	inpo,dsyn,phpranab,orgf …	-J in metamap. Semantic type abbrevation. Only including the specific semantic type terms in the result.
conjunction	True or False. Default is True	–conj in metamap. Metamap default chunks the input text into short phrase, this parameter means considering the input as one phrase.
term_processing	True or False. Default is True	-J in metamap. -z in metamap. Metamap will treat the input as one term. The function is similar with –conj.

- **source:** While parsing the same text, different vocabulary source could result in different MeSH terms. For example, the same text "Ataxias, Gait", when we used MeSH, UMLS or OMIM as vocabulary source separately, the parsed UMLS concept are "C0751837", "C0004134" and "C0751837". Its corresponding MeSH terms are "'D020234", 'D001259', "'D020234". As it showed, the results are inconsistent. pyMeSHSim accepted a list of thesaurus sources in text parsing, but we recommended to use the specific thesaurus source according the subject of one's research. The default is MSH, which means use MeSH as the specific semantic source.

```
>>> from pyMeSHSim.metamapWrap.MetamapInterface import MetaMap
>>> metamap = MetaMap(path="/home/luozhihui/Software/public_mm/bin/metamap16")
>>> concept = metamap.runMetaMap(semantic_types=metamap.semanticTypes, text="Ataxias,␣
↪Gait", source=["MSH"])
>>> print(concept)
[{'index': '00000000', 'mm': 'MMI', 'score': '20.95', 'preferred_name': 'Gait Ataxia',
↪ 'cui': 'C0751837', 'semtypes': '[sosy]', 'trigger': '["Gait Ataxia"-tx-1-"Ataxias␣
↪Gait"-noun-0]', 'location': 'TX', 'pos_info': '0/7,9/4', 'tree_codes': 'C10.597.350.
↪090.750;C10.597.404.450;C23.888.592.350.090.600;C23.888.592.413.450', 'MeSHID':
↪'D020234'}]
```

```
>>> concept = metamap.runMetaMap(semantic_types=metamap.semanticTypes, text="Ataxias,␣
↪Gait", source=["MTH"])
>>> print(concept)
[{'index': '00000000', 'mm': 'MMI', 'score': '16.26', 'preferred_name': 'Ataxia', 'cui
↪': 'C0004134', 'semtypes': '[sosy]', 'trigger': '["Ataxia"-tx-1-"Ataxias"-noun-0]',
↪'location': 'TX', 'pos_info': '0/7', 'tree_codes': 'C10.597.350.090;C23.888.592.350.
↪090', 'MeSHID': 'D001259'}]
```

```
>>> concept = metamap.runMetaMap(semantic_types=metamap.semanticTypes, text="Ataxias,␣
↪Gait", source=["OMIM"])
>>> print(concept)
[{'index': '00000000', 'mm': 'MMI', 'score': '20.95', 'preferred_name': 'Gait Ataxia',
↪ 'cui': 'C0751837', 'semtypes': '[sosy]', 'trigger': '["Gait ataxia"-tx-1-"Ataxias␣
↪Gait"-noun-0]', 'location': 'TX', 'pos_info': '0/7,9/4', 'tree_codes': 'C10.597.350.
↪090.750;C10.597.404.450;C23.888.592.350.090.600;C23.888.592.413.450', 'MeSHID':
↪'D020234'}]
```

- **semantic_types:** All MeSH main headings were belonged to some kinds of semantic types. Assigning some clear semantic types will be helpful when parsing the free text. For example, the text "breast, cancer", if we use 'neop' as semantic type, the parsed result was 'Malignant Neoplasms'. But when we use 'bpoc' as semantic type, the result was 'Breast'. The 'neop' and 'bpoc' means "Neoplastic Process" and "Body Part, Organ, or Organ Component" respectively. All semantic type abbreviations can be seen in the reference (https://metamap.nlm.nih.gov/Docs/SemanticTypes_2018AB.txt). The default semantic types were none, and all semantic would be considered. all disease related sematic types had been embedded in pyMeSH-

Sim, users can define the 'semantic_types' as 'metamap.semanticTypes' to restrict all parsed result to disease terms.

```
>>> concept = metamap.runMetaMap( text="breast, cancer", semantic_types=["neop"],␣
↪conjunction=False, term_processing=False )
>>> print(concept)
[{'index': '00000000', 'mm': 'MMI', 'score': '5.18', 'preferred_name': 'Malignant␣
↪Neoplasms', 'cui': 'C0006826', 'semtypes': '[neop]', 'trigger': '["Cancer"-tx-1-
↪"cancer"-noun-0]', 'location': 'TX', 'pos_info': '8/6', 'tree_codes': ['C04'],
↪'MeSHID': 'D009369'}]
```

```
>>> concept = metamap.runMetaMap( text="breast, cancer", semantic_types=["bpoc"],␣
↪conjunction=False, term_processing=False )
>>> print(concept)
[{'index': '00000000', 'mm': 'MMI', 'score': '8.34', 'preferred_name': 'Breast', 'cui
↪': 'C0006141', 'semtypes': '[bpoc]', 'trigger': '["Breast"-tx-1-"breast"-noun-0]',
↪'location': 'TX', 'pos_info': '0/6', 'tree_codes': 'A01.236', 'MeSHID': 'D001940'}]
```

- **Conjunction and term_processing:** The conjunction was new character of metamap from 2016 version 2. In fact, the conjunction and term_processing had similar functions, both would treat the input as one single phrase, will not chunked the input into separate text. While the conjunction is True and term_processing is True, the input text will be treated as one short phrase, when the conjunction is False and term_processing is "False", the input text will be treated as long sentence, and metamap will try to parse the UMLS concept from part of the sentence. For "Ataxias, Gait", when we set both parameters "True", the result is "'Gait Ataxia", but when we set them "False", the result would be 'Ataxia' and 'Gait' separately. In pyMeSHSim, if we are parsing a long sentence, we should turn the conjunction and term_processing to "False". When we are parsing a short sentence, we should turn the Conjunction and term_processing to "True". The metamap recommended to use term processing together with ignore_word_order. So, we default set the ignore_word_order on True as default.

```
>>> concept = metamap.runMetaMap( text="Ataxias, Gait", semantic_types=metamap.
↪semanticTypes)
>>> print(concept)
[{'index': '00000000', 'mm': 'MMI', 'score': '20.95', 'preferred_name': 'Gait Ataxia',
↪ 'cui': 'C0751837', 'semtypes': '[sosy]', 'trigger': '["Gait Ataxia"-tx-1-"Ataxias␣
↪Gait"-noun-0]', 'location': 'TX', 'pos_info': '0/7,9/4', 'tree_codes': 'C10.597.350.
↪090.750;C10.597.404.450;C23.888.592.350.090.600;C23.888.592.413.450', 'MeSHID':
↪'D020234'}]
```

```
>>> concept = metamap.runMetaMap( text="Ataxias, Gait", semantic_types=metamap.
↪semanticTypes, conjunction=False, term_processing=False)
>>> print(concept)
[{'index': '00000000', 'mm': 'MMI', 'score': '17.80', 'preferred_name': 'Ataxia', 'cui
↪': 'C0004134', 'semtypes': '[sosy]', 'trigger': '["Ataxia"-tx-1-"Ataxias"-noun-0]',
↪'location': 'TX', 'pos_info': '0/7', 'tree_codes': 'C10.597.350.090;C23.888.592.350.
↪090', 'MeSHID': 'D001259'}, {'index': '00000000', 'mm': 'MMI', 'score': '17.80',
↪'preferred_name': 'Gait', 'cui': 'C0016928', 'semtypes': '[fndg]', 'trigger': '[
↪"Gait"-tx-1-"Gait"-noun-0]', 'location': 'TX', 'pos_info': '9/4', 'tree_codes':
↪'E01.370.600.250;G11.427.590.530.389', 'MeSHID': 'D005684'}]
```

The other parameters in metamapWrap are format options. And detail information can be seen in reference.

3.1.2 Data

The data subpackage contained the basic data frame in pyMeSHSim. It had two major functions, constructing MeSH data from UMLS metathesaurus and establishing data application interfaces. The data construction module made the

data update easily. And the data application interface module made it possible to be used by other developers. Based on the data subpackage, pyMeSHSim provided a series of useful function as descripted in extended table 2. Application interface Duction description

Table 2: library function in data

function name	Description
getMeSHConcept	Obtaining MeSH terms from UMLS concepts
getMeSHConcept	Obtaining MeSH term detail from MeSH ID
getUMLSIDbyMeSHID	Obtaining UMLS concept from MeSH ID
getCategory	Obtaining the category of MeSH terms
convertToNarrow	Obtaining narrow or broad terms of a mesh term
getParentsConceptID	Obtaining parent or child terms of a MeSH term
getTopConceptID	Obtaining the top term of a MeSH term
getAncestors	Obtaining the MeSH ID by MeSH tree code
getPrefferedName	Obtaining the preffered name by MeSH ID

The examples of these interfaces could be seen in https://pymeshsim.readthedocs.io/en/latest/tutorial.html#term-library.

3.1.3 Sim

PyMeSHSim had implemented five semantic similarity algorithms. Four IC-based algorithms and one graph-based algorithm. The IC-base methods had fixed Algorithm formulas, but the graph-based method could be influenced by a parameter ω weight. We test the ω weight on the GWAS phenotypes. For the MeSH term pairs parsed by Nelson's group manual work and pyMeSHSim, we calculated the semantic similarity between them. The result was displayed in supplementary table 4. We excluded the term pairs, which had sematic similarities equal to 1 or 0 in all algorithms.

Then, we investigated the increment of semantic similarity when increasing the weight by a step 0.1. As the supplementary figure 1A showed, the semantic similarity increase stably while weight increase. So, if users use Wang's method to measure the semantic similarity, any weight ω could distinguish MeSH term's difference in the same extent. To be noticed, different ω is suit for different threshold that indicating a pair of MeSH terms are similar. If comparing this method to IC-based methods, ω 0.6 is recommended. As it has a highest correlation with other algorithms compared to other values. (Supplementary figure 1B)

3.2 pipeline script

As pyMeSHSim implemented bio-NE recognition, normalization, and comparison functions. To make it easily used, we provide an example script "pipeline.py" to do batch processing.

The example commands were as below: Text parsing:

```
$ ./pipeline.py textParse /home/luozhihui/Software/public_mm/bin/metamap16 /home/
↪luozhihui/Project/free_text.reFormat_1.txt /home/luozhihui/Project/output_dir --
↪source=MSH -short
```

The input file need to be separated by "|", and it only has two columns.

Table 3: input file one

1|Serum uric acid
2|Nephrolithiasis
3|Non-albumin protein levels
4|Sphingolipid concentrations
5|Age-related macular degeneration
6|Systemic lupus erythematosus
7|Eye color traits
8|Type 1 diabetes autoantibodies
9|Type 1 diabetes
10|Systolic blood pressure

We had stored an example of this input file as "free_text.reFormat.txt" in pyMeSHSim.

Similarity calculating:

```
$ ./pipeline.py simCal ./output_dir/MeSH_term_pair.txt ./output_dir --
↪weight=0.7
```

The input file should be MeSH ID pairs, separated by tab. Just like below:

Table 4: input file two

D018932 D018945
D008589 D008585
D051436 D007676
D000418 D012709
D006526 D019698
D051436 D007676
D049971 D006852
D008548 D010859
D008548 D010859
D003176 D003165

We had stored an example of this input file as "MeSH_term_pair.txt" in pyMeSHSim.

3.3 Parsing free text

3.3.1 Parsing short sentence

We can access MetaMap with:

```
>>> from pyMeSHSim.metamapWrap.MetamapInterface import MetaMap
>>> metamap = MetaMap(path="/home/luozhihui/Project/UMLS/public_mm/bin/metamap16")
>>> concept = metamap.runMetaMap(semantic_types=metamap.semanticTypes, text="Ataxias,␣
↪Gait")
```

(continues on next page)

(continued from previous page)

```
>>> print(concept)
[{'index': '00000000', 'mm': 'MMI', 'score': '20.95', 'preferred_name': 'Gait Ataxia',
↪ 'cui': 'C0751837', 'semtypes': '[sosy]', 'trigger': '["Gait Ataxia"-tx-1-"Ataxias␣
↪Gait"-noun-0]', 'location': 'TX',
'pos_info': '0/7,9/4', 'tree_codes': 'C10.597.350.090.750;C10.597.404.450;C23.888.592.
↪350.090.600;C23.888.592.413.450', 'MeSHID': 'D020234'}]
```

3.3.2 Parsing long sentence

When parsing a long sentence, we should turn off the parameters "conjunction", "term_processing" as following:

```
>>> from pyMeSHSim.metamapWrap.MetamapInterface import MetaMap
>>> metamap = MetaMap(path="/home/luozhihui/Project/UMLS/public_mm/bin/metamap16")
>>> concept = metamap.runMetaMap(semantic_types=metamap.semanticTypes ,␣
↪conjunction=False, term_processing=False, text="131-I-TM-601 is investigated in␣
↪clinical trials for treating brain cancer. 131-I-TM-601 is a solid. Tx binds to and␣
↪reduces the activity of a matrix metalloproteinase (MMP) that regulates functioning␣
↪of the chloride channels on cell membranes. TM-601 is a small 36-amino-acid peptide␣
↪that selectively binds to glioma cells but not normal brain parenchyma. It is a␣
↪synthetic version of a neurotoxin isolated from the venom of the Giant Yellow␣
↪Israeli scorpion Leiurus quinquestriatus. The synthetic version of this peptide has␣
↪been manufactured and covalently linked to iodine 131 ((131)I-TM-601) as a means of␣
↪targeting radiation to tumor cells in the treatment of brain cancer. The selective␣
↪effects of TM-601 are regulated by its action on MMP2 receptors.")
>>> for con in concept:
>>>     print(con)
{'index': '00000000', 'mm': 'MMI', 'score': '19.34', 'preferred_name': 'Glioma', 'cui
↪': 'C0017638', 'semtypes': '[neop]', 'trigger': '["Glioma"-tx-4-"glioma"-noun-0]',
↪'location': 'TX', 'pos_info': '310/6', 'tree_codes': 'C04.557.465.625.600.380;C04.
↪557.470.670.380;C04.557.580.625.600.380', 'MeSHID': 'D005910'}
{'index': '00000000', 'mm': 'MMI', 'score': '16.22', 'preferred_name': 'Brain␣
↪Neoplasms', 'cui': 'C0006118', 'semtypes': '[neop]', 'trigger': '["Brain Neoplasms"-
↪tx-6-"tumor the of brain"-noun-0]', 'location': 'TX', 'pos_info': '633/5,648/3,662/8
↪', 'tree_codes': 'C04.588.614.250.195;C10.228.140.211;C10.551.240.250', 'MeSHID':
↪'D001932'}
{'index': '00000000', 'mm': 'MMI', 'score': '13.00', 'preferred_name': 'Gigantism',
↪'cui': 'C0017547', 'semtypes': '[dsyn]', 'trigger': '["Gigantism"-tx-5-"Giant"-adj-
↪0]', 'location': 'TX', 'pos_info': '429/5', 'tree_codes': 'C05.116.099.492;C05.116.
↪132.479;C19.700.355.528', 'MeSHID': 'D005877'}
{'index': '00000000', 'mm': 'MMI', 'score': '12.89', 'preferred_name':
↪'Electromagnetic Radiation', 'cui': 'C0034519', 'semtypes': '[npop]', 'trigger': '[
↪"Electromagnetic Radiation"-tx-6-"radiation"-noun-0]', 'location': 'TX', 'pos_info
↪': '620/9', 'tree_codes': 'G01.358.500.505;G01.750.250', 'MeSHID': 'D060733'}
{'index': '00000000', 'mm': 'MMI', 'score': '10.06', 'preferred_name': 'Plasma␣
↪membrane', 'cui': 'C0007603', 'semtypes': '[celc]', 'trigger': '["Plasma Membrane"-
↪tx-3-"cell membranes"-noun-0]', 'location': 'TX', 'pos_info': '228/14', 'tree_codes
↪': 'A11.284.149', 'MeSHID': 'D002462'}
{'index': '00000000', 'mm': 'MMI', 'score': '9.92', 'preferred_name': 'Brain', 'cui':
↪'C0006104', 'semtypes': '[bpoc]', 'trigger': '["Brain"-tx-4-"brain"-noun-0]',
↪'location': 'TX', 'pos_info': '338/5', 'tree_codes': 'A08.186.211', 'MeSHID':
↪'D001921'}
{'index': '00000000', 'mm': 'MMI', 'score': '9.69', 'preferred_name': 'Genetic␣
↪Selection', 'cui': 'C0036576', 'semtypes': '[genf]', 'trigger': '["Genetic Selection
↪"-tx-7-"selective"-adj-0]', 'location': 'TX', 'pos_info': '683/9', 'tree_codes':
↪'G05.355.800', 'MeSHID': 'D012641'}
```

(continues on next page)

(continued from previous page)

```
{'index': '00000000', 'mm': 'MMI', 'score': '7.17', 'preferred_name': 'Malignant␣
↪neoplasm of brain', 'cui': 'C0153633', 'semtypes': '[neop]', 'trigger': '["Brain␣
↪Neoplasm, Malignant"-tx-6-"the of brain cancer"-noun-0,"Brain Neoplasm, Malignant"-
↪tx-1-"brain cancer"-noun-0]', 'location': 'TX', 'pos_info': '648/3,662/15;61/12',
↪'tree_codes': '', 'MeSHID': None}
{'index': '00000000', 'mm': 'MMI', 'score': '7.15', 'preferred_name': 'Cells', 'cui':
↪'C0007634', 'semtypes': '[cell]', 'trigger': '["Cells"-tx-6-"cells"-noun-0,"Cells"-
↪tx-4-"cells"-noun-0]', 'location': 'TX', 'pos_info': '639/5;317/5', 'tree_codes':
↪'A11', 'MeSHID': 'D002477'}
{'index': '00000000', 'mm': 'MMI', 'score': '6.58', 'preferred_name': 'Radiation',
↪'cui': 'C0851346', 'semtypes': '[npop]', 'trigger': '["Radiation"-tx-6-"radiation"-
↪noun-0]', 'location': 'TX', 'pos_info': '620/9', 'tree_codes': 'G01.750', 'MeSHID':
↪'D011827'}
{'index': '00000000', 'mm': 'MMI', 'score': '3.54', 'preferred_name': 'Neoplasms',
↪'cui': 'C0027651', 'semtypes': '[neop]', 'trigger': '["Neoplasms"-tx-6-"tumor"-noun-
↪0]', 'location': 'TX', 'pos_info': '633/5', 'tree_codes': 'C04', 'MeSHID': 'D009369
↪'}
{'index': '00000000', 'mm': 'MMI', 'score': '3.42', 'preferred_name': 'Malignant␣
↪Neoplasms', 'cui': 'C0006826', 'semtypes': '[neop]', 'trigger': '["Cancer"-tx-6-
↪"cancer"-noun-0]', 'location': 'TX', 'pos_info': '671/6', 'tree_codes': '', 'MeSHID
↪': None}
```

3.3.3 Filtering the result

While parsing a long sentence, we will get many results. We can discard the ancestor concepts.

```
>>> from pyMeSHSim.Sim.similarity import metamapFilter
>>> filter = metamapFilter(path="/home/luozhihui/Project/UMLS/public_mm/bin/metamap16
↪")
>>> concepts = filter.runMetaMap(semantic_types=filter.semanticTypes ,␣
↪conjunction=False, term_processing=False, text="131-I-TM-601 is investigated in␣
↪clinical trials for treating brain cancer. 131-I-TM-601 is a solid. Tx binds to and␣
↪reduces the activity of a matrix metalloproteinase (MMP) that regulates functioning␣
↪of the chloride channels on cell membranes. TM-601 is a small 36-amino-acid peptide␣
↪that selectively binds to glioma cells but not normal brain parenchyma. It is a␣
↪synthetic version of a neurotoxin isolated from the venom of the Giant Yellow␣
↪Israeli scorpion Leiurus quinquestriatus. The synthetic version of this peptide has␣
↪been manufactured and covalently linked to iodine 131 ((131)I-TM-601) as a means of␣
↪targeting radiation to tumor cells in the treatment of brain cancer. The selective␣
↪effects of TM-601 are regulated by its action on MMP2 receptors.")
>>> results = filter.discardAncestor(concepts=concepts)
>>> for res in results:
>>>     print (res)
{'index': '00000000', 'mm': 'MMI', 'score': '6.58', 'preferred_name': 'Radiation',
↪'cui': 'C0851346', 'semtypes': '[npop]', 'trigger': '["Radiation"-tx-6-"radiation"-
↪noun-0]', 'location': 'TX', 'pos_info': '620/9', 'tree_codes': 'G01.750', 'MeSHID':
↪'D011827'}
{'index': '00000000', 'mm': 'MMI', 'score': '9.69', 'preferred_name': 'Genetic␣
↪Selection', 'cui': 'C0036576', 'semtypes': '[genf]', 'trigger': '["Genetic Selection
↪"-tx-7-"selective"-adj-0]', 'location': 'TX', 'pos_info': '683/9', 'tree_codes':
↪'G05.355.800', 'MeSHID': 'D012641'}
{'index': '00000000', 'mm': 'MMI', 'score': '16.22', 'preferred_name': 'Brain␣
↪Neoplasms', 'cui': 'C0006118', 'semtypes': '[neop]', 'trigger': '["Brain Neoplasms"-
↪tx-6-"tumor the of brain"-noun-0]', 'location': 'TX', 'pos_info': '633/5,648/3,662/8
↪', 'tree_codes': 'C04.588.614.250.195;C10.228.140.211;C10.551.240.250', 'MeSHID':
↪'D001932'}
```

(continues on next page)

(continued from previous page)

```
{'index': '00000000', 'mm': 'MMI', 'score': '13.00', 'preferred_name': 'Gigantism',
↪'cui': 'C0017547', 'semtypes': '[dsyn]', 'trigger': '["Gigantism"-tx-5-"Giant"-adj-
↪0]', 'location': 'TX', 'pos_info': '429/5', 'tree_codes': 'C05.116.099.492;C05.116.
↪132.479;C19.700.355.528', 'MeSHID': 'D005877'}
{'index': '00000000', 'mm': 'MMI', 'score': '7.15', 'preferred_name': 'Cells', 'cui':
↪'C0007634', 'semtypes': '[cell]', 'trigger': '["Cells"-tx-6-"cells"-noun-0,"Cells"-
↪tx-4-"cells"-noun-0]', 'location': 'TX', 'pos_info': '639/5;317/5', 'tree_codes':
↪'A11', 'MeSHID': 'D002477'}
{'index': '00000000', 'mm': 'MMI', 'score': '9.92', 'preferred_name': 'Brain', 'cui':
↪'C0006104', 'semtypes': '[bpoc]', 'trigger': '["Brain"-tx-4-"brain"-noun-0]',
↪'location': 'TX', 'pos_info': '338/5', 'tree_codes': 'A08.186.211', 'MeSHID':
↪'D001921'}
{'index': '00000000', 'mm': 'MMI', 'score': '12.89', 'preferred_name':
↪'Electromagnetic Radiation', 'cui': 'C0034519', 'semtypes': '[npop]', 'trigger': '[
↪"Electromagnetic Radiation"-tx-6-"radiation"-noun-0]', 'location': 'TX', 'pos_info
↪': '620/9', 'tree_codes': 'G01.358.500.505;G01.750.250', 'MeSHID': 'D060733'}
{'index': '00000000', 'mm': 'MMI', 'score': '19.34', 'preferred_name': 'Glioma', 'cui
↪': 'C0017638', 'semtypes': '[neop]', 'trigger': '["Glioma"-tx-4-"glioma"-noun-0]',
↪'location': 'TX', 'pos_info': '310/6', 'tree_codes': 'C04.557.465.625.600.380;C04.
↪557.470.670.380;C04.557.580.625.600.380', 'MeSHID': 'D005910'}
{'index': '00000000', 'mm': 'MMI', 'score': '3.54', 'preferred_name': 'Neoplasms',
↪'cui': 'C0027651', 'semtypes': '[neop]', 'trigger': '["Neoplasms"-tx-6-"tumor"-noun-
↪0]', 'location': 'TX', 'pos_info': '633/5', 'tree_codes': 'C04', 'MeSHID': 'D009369
↪'}
{'index': '00000000', 'mm': 'MMI', 'score': '10.06', 'preferred_name': 'Plasma␣
↪membrane', 'cui': 'C0007603', 'semtypes': '[celc]', 'trigger': '["Plasma Membrane"-
↪tx-3-"cell membranes"-noun-0]', 'location': 'TX', 'pos_info': '228/14', 'tree_codes
↪': 'A11.284.149', 'MeSHID': 'D002462'}
```

We can also discard general MeSH terms with too many descendants.

```
>>> from pyMeSHSim.Sim.similarity import metamapFilter
>>> filter = metamapFilter(path="/home/luozhihui/Project/UMLS/public_mm/bin/metamap16
↪")
>>> concepts = filter.runMetaMap(semantic_types=filter.semanticTypes ,␣
↪conjunction=False, term_processing=False, text="131-I-TM-601 is investigated in␣
↪clinical trials for treating brain cancer. 131-I-TM-601 is a solid. Tx binds to and␣
↪reduces the activity of a matrix metalloproteinase (MMP) that regulates functioning␣
↪of the chloride channels on cell membranes. TM-601 is a small 36-amino-acid peptide␣
↪that selectively binds to glioma cells but not normal brain parenchyma. It is a␣
↪synthetic version of a neurotoxin isolated from the venom of the Giant Yellow␣
↪Israeli scorpion Leiurus quinquestriatus. The synthetic version of this peptide has␣
↪been manufactured and covalently linked to iodine 131 ((131)I-TM-601) as a means of␣
↪targeting radiation to tumor cells in the treatment of brain cancer. The selective␣
↪effects of TM-601 are regulated by its action on MMP2 receptors.")
>>> results = filter.discardNodeHigh(number=50, concepts=concepts)
>>> for res in results:
>>>     print (res)
{'index': '00000000', 'mm': 'MMI', 'score': '19.34', 'preferred_name': 'Glioma', 'cui
↪': 'C0017638', 'semtypes': '[neop]', 'trigger': '["Glioma"-tx-4-"glioma"-noun-0]',
↪'location': 'TX', 'pos_info': '310/6', 'tree_codes': 'C04.557.465.625.600.380;C04.
↪557.470.670.380;C04.557.580.625.600.380', 'MeSHID': 'D005910'}
{'index': '00000000', 'mm': 'MMI', 'score': '16.22', 'preferred_name': 'Brain␣
↪Neoplasms', 'cui': 'C0006118', 'semtypes': '[neop]', 'trigger': '["Brain Neoplasms"-
↪tx-6-"tumor the of brain"-noun-0]', 'location': 'TX', 'pos_info': '633/5,648/3,662/8
↪', 'tree_codes': 'C04.588.614.250.195;C10.228.140.211;C10.551.240.250', 'MeSHID':
↪'D001932'}
```

(continues on next page)

(continued from previous page)

```
{'index': '00000000', 'mm': 'MMI', 'score': '13.00', 'preferred_name': 'Gigantism',
↪'cui': 'C0017547', 'semtypes': '[dsyn]', 'trigger': '["Gigantism"-tx-5-"Giant"-adj-
↪0]', 'location': 'TX', 'pos_info': '429/5', 'tree_codes': 'C05.116.099.492;C05.116.
↪132.479;C19.700.355.528', 'MeSHID': 'D005877'}
{'index': '00000000', 'mm': 'MMI', 'score': '12.89', 'preferred_name':
↪'Electromagnetic Radiation', 'cui': 'C0034519', 'semtypes': '[npop]', 'trigger': '[
↪"Electromagnetic Radiation"-tx-6-"radiation"-noun-0]', 'location': 'TX', 'pos_info
↪': '620/9', 'tree_codes': 'G01.358.500.505;G01.750.250', 'MeSHID': 'D060733'}
{'index': '00000000', 'mm': 'MMI', 'score': '10.06', 'preferred_name': 'Plasma␣
↪membrane', 'cui': 'C0007603', 'semtypes': '[celc]', 'trigger': '["Plasma Membrane"-
↪tx-3-"cell membranes"-noun-0]', 'location': 'TX', 'pos_info': '228/14', 'tree_codes
↪': 'A11.284.149', 'MeSHID': 'D002462'}
{'index': '00000000', 'mm': 'MMI', 'score': '9.69', 'preferred_name': 'Genetic␣
↪Selection', 'cui': 'C0036576', 'semtypes': '[genf]', 'trigger': '["Genetic Selection
↪"-tx-7-"selective"-adj-0]', 'location': 'TX', 'pos_info': '683/9', 'tree_codes':
↪'G05.355.800', 'MeSHID': 'D012641'}
{'index': '00000000', 'mm': 'MMI', 'score': '7.17', 'preferred_name': 'Malignant␣
↪neoplasm of brain', 'cui': 'C0153633', 'semtypes': '[neop]', 'trigger': '["Brain␣
↪Neoplasm, Malignant"-tx-6-"the of brain cancer"-noun-0,"Brain Neoplasm, Malignant"-
↪tx-1-"brain cancer"-noun-0]', 'location': 'TX', 'pos_info': '648/3,662/15;61/12',
↪'tree_codes': ['C10.551.240.250', 'C10.228.140.211', 'C04.588.614.250.195'], 'MeSHID
↪': 'D001932'}
{'index': '00000000', 'mm': 'MMI', 'score': '6.58', 'preferred_name': 'Radiation',
↪'cui': 'C0851346', 'semtypes': '[npop]', 'trigger': '["Radiation"-tx-6-"radiation"-
↪noun-0]', 'location': 'TX', 'pos_info': '620/9', 'tree_codes': 'G01.750', 'MeSHID':
↪'D011827'}
```

3.4 Term library

Apart from metamapWrap, class termComp inherits all the class in pyMeSHSim, and thus many functions can be invoked by termComp.

3.4.1 Obtaining MeSH terms from UMLS concepts

```
>>> from pyMeSHSim.Sim.similarity import termComp
>>> simCom = termComp()
>>> concept = simCom.getMeSHConcept(cui="C0024116")
>>> print(concept)
{'cui': 'C0024116', 'MeSHID': 'D008172', 'semtypes': 'dsyn', 'tree_code': ['C08.730.
↪435', 'C01.703.534', 'C08.381.472'], 'preferred_name': 'Fungal Lung Disease'}
```

3.4.2 Obtaining MeSH term detail from MeSH ID

This method will be helpful.

```
>>> from pyMeSHSim.Sim.similarity import termComp
>>> simCom = termComp()
>>> concept = simCom.getMeSHConcept(dui="D008674")
```

(continues on next page)

(continued from previous page)

```
>>> print(concept)
{'cui': 'C0025556', 'MeSHID': 'D008674', 'semtypes': 'elii', 'tree_code': ['D01.268.
↪558', 'D01.552.550'], 'preferred_name': 'Earth Metals, Rare'}
```

3.4.3 Obtaining UMLS concept from MeSH ID

If one MeSH record corresponds to more than one UMLS concepts, pyMeSHSim only provides the recommended one.

```
>>> from pyMeSHSim.Sim.similarity import termComp
>>> simCom = termComp()
>>> con = simCom.getUMLSIDbyMeSHID(dui="D008674")
>>> print (con)
C0025556
```

3.4.4 Obtaining the category of MeSH terms

A lot of processing should denote the category of terms, it is necessary to get it in the begining.
getCategory will return the category of a term.

```
>>> from pyMeSHSim.Sim.similarity import termComp
>>> simCom = termComp()
>>> ty = simCom.getCategory(dui="D008674")
>>> print (ty)
['D']
>>>ty = simCom.getCategory(dui="D008674")
>>>print (ty)
['F']
```

3.4.5 Obtaining narrow or broad terms of a mesh term

We can obtain the narrow terms of main headings by convertToNarrow and obtain the broad terms of SCRs by convertToBroad.

```
>>> from pyMeSHSim.Sim.similarity import termComp
>>> simCom = termComp()
>>> NRs = simCom.convertToNarrow(dui="D000544")
>>> print (Nrs)
['C564330', 'C565078', 'C565228', 'C563834', 'C565251', 'C565325', 'C567463', 'C566465
↪', 'C567022', 'C566999', 'C536595', 'C566998', 'C566578', 'C563254', 'C536596',
↪'C564622', 'C536594', 'C566298', 'C567000', 'C564329', 'C566299', 'C565728',
↪'C536598', 'C536599']
>>> BRs = simCom.convertToBroad(dui="C565078")
>>> print (BRs)
['D000544']
```

3.4.6 Obtaining parent or child terms of a MeSH term

We can obtain child and parent terms of main headings by getParentsConceptID and getChildrenConceptID, respectively.

```
>>> from pyMeSHSim.Sim.similarity import termComp
>>> simCom = termComp()
>>> parents = simCom.getParentsConceptID(dui="D000544", category="C")
>>> print (parents)
['D024801', 'D003704']
>>> children = simCom.getChildrenConceptID(dui="D012559", category="F")
>>> print (children)
['D012753', 'D012562', 'D012563', 'D012560']
```

3.4.7 Obtaining the top term of a MeSH term

We can obtain the top term of a MeSH term by getTopConceptID.

```
>>> from pyMeSHSim.Sim.similarity import termComp
>>> simCom = termComp()
>>> topTerm = simCom.getTopConceptID(dui="D000544")
>>> print (topTerm)
{'D001523': 'Disorders, Mental', 'D009422': 'Diseases, Nervous System'}
```

3.4.8 Obtaining the ancestors or offsprings

We can obtain ancestors or offsprings of main headings by getAncestors or getDescendant, respectively.

```
>>> from pyMeSHSim.Sim.similarity import termComp
>>> simCom = termComp()
>>> ancestors = simCom.getAncestors(dui="D000544", category="C")
>>> print (ancestors)
['D000544', 'D024801', 'D003704', 'D019636', 'D001927', 'D009422', 'D002493']
>>> descendant = simCom.getDescendant(dui="D012559", category="F")
>>> print (descendant)
['D012559', 'D012563', 'D012753', 'D012562', 'D012560']
```

3.4.9 Retrieving MeSH ID by MeSH tree code

```
>>> from pyMeSHSim.Sim.similarity import termComp
>>> simCom = termComp()
>>> dui = simCom.getDuiFromTreeCode(treeCode="D01.552.550")
>>> print(dui)
D008674
```

3.4.10 Obtaining the preffered name by MeSH ID

We can obtain the preffered name of MeSH terms by getPrefferedName.

```
>>> from pyMeSHSim.Sim.similarity import termComp
>>> simCom = termComp()
>>> name = simCom.getPrefferedName(dui="D008674")
>>> print(name)
Earth Metals, Rare
```

3.5 Calculating similarity

3.5.1 Calculating similarity between MeSH terms

We can calculate semantic similarity between MeSH terms with five algorithms [“lin”, “res”, “jiang”, “rel”, “wang”].

```
>>> from pyMeSHSim.Sim.similarity import termComp
>>> simCom = termComp()
>>> #in different category, the similarity will be different
>>> simCom.termSim(dui1="D000544", dui2="D006816", method="lin", category="F")
D000544 D006816 0.6957847588446384
>>> simCom.termSim(dui1="D000544", dui2="D006816", method="lin", category="C")
D000544 D006816 0.7500806317732097
```

```
>>> from pyMeSHSim.Sim.similarity import termComp
>>> simCom = termComp()
>>> #between mainheading and SCR, IC method
>>> simCom.termSim(dui1="C565251", dui2="D012559", method="rel", category="F")
C565251 D012559 0.3314002496646189
>>> #based on path
>>> simCom.termSim(dui1="C565251", dui2="D012559", method="wang", category="F")
C565251 D012559 0.17637095066694894
```

CHAPTER 4

Library reference

4.1 data

`pyMeSHSim.data.createData`
`pyMeSHSim.data.dataInterface`
`pyMeSHSim.data.duiFunc`

4.1.1 pyMeSHSim.data.createData

4.1.2 pyMeSHSim.data.dataInterface

4.1.3 pyMeSHSim.data.duiFunc

4.2 metamapWrap

`pyMeSHSim.metamapWrap.Concept`
`pyMeSHSim.metamapWrap.`<br>`MetamapInterface`

4.2.1 pyMeSHSim.metamapWrap.Concept

4.2.2 pyMeSHSim.metamapWrap.MetamapInterface

4.3 Sim

`pyMeSHSim.Sim.ICScore`
`pyMeSHSim.Sim.PathScore`
`pyMeSHSim.Sim.MeSHProcess`
`pyMeSHSim.Sim.similarity`

4.3.1 pyMeSHSim.Sim.ICScore

4.3.2 pyMeSHSim.Sim.PathScore

4.3.3 pyMeSHSim.Sim.MeSHProcess

4.3.4 pyMeSHSim.Sim.similarity

CHAPTER 5

Indices and tables

- genindex
- modindex
- search
